# Breast Cancer “Tailored Follow-up” in Italian Oncology Units: A Web-Based Survey

**DOI:** 10.1371/journal.pone.0094063

**Published:** 2014-04-08

**Authors:** Clara Natoli, Davide Brocco, Isabella Sperduti, Antonio Nuzzo, Nicola Tinari, Michele De Tursi, Antonino Grassadonia, Lorenzo Mazzilli, Stefano Iacobelli, Teresa Gamucci, Patrizia Vici, the “FOLLOW-UP” Study Group

**Affiliations:** 1 Department of Experimental and Clinical Sciences, University “G. d'Annunzio”, Chieti, Italy; 2 Unit of Biostatistics, Regina Elena National Cancer Institute, Rome, Italy; 3 Oncology Department, “Floraspe Renzetti” Hospital, Lanciano, Italy; 4 Clinical Governance Unit, “SS. Annunziata” Hospital, Chieti, Italy; 5 Department of Oncology, “S.S. Trinita′” Hospital, Sora, Italy; 6 Division of Medical Oncology B, Regina Elena National Cancer Institute, Rome, Italy; Health Canada and University of Ottawa, Canada

## Abstract

**Purpose:**

Breast cancer follow-up procedures after primary treatment are still a controversial issue. Aim of this study was to investigate, through a web-based survey, surveillance methodologies selected by Italian oncologists in everyday clinical practice.

**Methods:**

Referents of Italian medical oncology units were invited to participate to the study via e-mail through the SurveyMonkey website. Participants were asked how, in their institution, exams of disease staging and follow-up are planned in asymptomatic women and if surveillance continues beyond the 5^th^ year.

**Results:**

Between February and May 2013, 125 out of 233 (53.6%) invited referents of Italian medical oncology units agreed to participate in the survey. Ninety-seven (77.6%) referents state that modalities of breast cancer follow-up are planned according to the risk of disease progression at diagnosis and only 12 (9.6%) oncology units apply the minimal follow-up procedures according to international guidelines. Minimal follow-up is never applied in high risk asymptomatic women. Ninety-eight (78.4%) oncology units continue follow-up in all patients beyond 5 years.

**Conclusions:**

Our survey shows that 90.4% of participating Italian oncology units declare they do not apply the minimal breast cancer follow-up procedures after primary treatment in asymptomatic women, as suggested by national and international guidelines. Interestingly, about 80.0% of interviewed referents performs the so called “tailored follow-up”, high intensity for high risk, low intensity for low risk patients. There is an urgent need of randomized clinical trials able to determine the effectiveness of risk-based follow-up modalities, their ideal frequency and persistence in time.

## Introduction

Breast cancer surveillance procedures after primary treatment are common practice in clinical oncology even if their methodology is still a controversial issue. The primary aim of breast cancer follow-up is to decrease mortality and improve well-being through early detection of second ipsi- or contralateral cancer and local recurrences, which are potentially curable, and ascertainment of symptoms suggestive of metastatic disease [1]. On the other hand, detection of metastatic disease in asymptomatic patients by intensive surveillance including complete blood counts, chemistry panels, tumor markers, imaging modalities (i.e. chest radiographs, bone scans, liver ultrasound and others) has not been demonstrated to improve overall survival [2], [3], [4], [5]. The secondary aim of breast cancer follow-up is diagnosis and management of morbidity due to adjuvant therapies [6], as well as improvement of adherence to endocrine therapy [7], [8] and assistance for psycho-social support [5].

Since the first release of breast cancer follow-up guidelines for management of early breast cancer patients in 1997 by the American Society of Clinical Oncology (ASCO) [9], recommending minimal follow-up procedures (i.e. regular history taking, clinical examination, annual surveillance mammography and breast self-examination), this topic has been of great interest for clinical oncologists [5], [10]. ASCO breast cancer follow-up guidelines have been periodically updated and similar recommendations have been adopted worldwide [11], [12], [13], [14], [15], [16], [17]. In Italy, breast cancer guidelines by the Associazione Italiana di Oncologia Medica (AIOM) recommend yearly mammography, self breast examination, visit for history and physical examination, genetic counseling as appropriate, gynecological visit, gynecological echography and pap test [18]. Blood lipid profile and bone density scan are suggested for women treated with adjuvant aromatase inhibitors. Other blood and imaging examinations are not advised in asymptomatic patients. Visits should be performed every 3–6 months for years 1–3, every 6–12 months for years 4–5, and annually thereafter.

Even if data from randomized clinical trials and extensive revisions of literature [2], [3], [4], [5], [19] are strongly suggestive that intensive follow-up does not improve survival or life, in clinical practice breast cancer patients are frequently addressed to this modality of surveillance [8], [20], [21], [22], [23], [24], [25]. The great improvements made over the last ten years in imaging modalities and therapies have prompted the need for more intensive procedures than those suggested by guidelines. Still open questions are those related to the choice of the best test to be applied, the optimal monitoring frequency and the duration of controls after primary surgery [11], while there is a general agreement both on annual surveillance mammography and on tests to be applied for early diagnosis and management of morbidity due to adjuvant therapies.

Aim of this study has been to investigate, through a web-based survey, which follow-up procedures are selected by Italian oncologists in everyday clinical practice, besides those universally accepted, such as annual mammography and adjuvant therapies related toxicities monitoring.

## Materials and Methods

### Ethics statement

As the study did not involve human subjects and no patient data were collected, ethics approval was not required.

### Participants

Referents of Italian medical oncology units were invited to participate to the study via e-mail through the SurveyMonkey website between January and May 2013. One recall was sent out by e-mail after one month from the first request.

Participants were asked to complete a first page with their personal data, name, surname, institution, address, city. Then they were required to answer if, in their institution, exams of disease staging and follow-up are performed in a similar way for all asymptomatic breast cancer patients or are stratified according to the risk of disease progression, classified as low or high. If the answer was “NO”, they were asked if, always in asymptomatic women, blood chemistry tests, tumor markers, chest radiograph, liver ultrasound, bone scan, whole-body CT scan, whole-body PET/CT scan are performed at diagnosis and at follow-up, and, if yes, how many times/year from year 1 to 5 after primary surgical treatment. If participants declared to perform follow-up surveillance according to the risk of disease progression, they were asked to choose which of the following factors they deem more relevant to classify patients at high risk (more options allowed): Luminal B/HER2-, Luminal B/HER2+, HER2+, Triple negative, pT2, pT3, pT4, pN1, pN2, pN3 or others to be specified. Then they were asked if they carry out blood exams, tumor markers, chest radiography, liver ultrasound, bone scan, whole-body CT scan, whole-body PET/CT scan at diagnosis and at follow-up in low and high risk categories, and, if yes, how many times/year from year 1 to 5 in both groups. Independently on how follow-up was performed, all participants were finally asked if they continue follow-up beyond the 5^th^ year with 3 responses to be selected: no, yes, only in estrogen receptor (ER) positive (**+**) patients.

All participating Medical Oncology Units were informed that the results of the study were going to be published and requirements for authorship was clearly indicated. There was no need to protect details of the participants since these were not patients and no personal data was collected. No patient data were collected, so ethics approval was not required.

### Statistical analysis

Data were analyzed through descriptive statistics. Differences in proportions and comparisons between groups were performed by using the chi-square or Fisher's exact test when appropriate. Due to a nonparametric distribution, data on frequency of exams during follow-up were compared with the Friedman Test followed by the Dunn's Multiple Comparison Test A p value below 0.05 was retained as statistically significant. SPSS software (SPSS version 21.0, SPSS Inc., Chicago, Illinois, USA) was used for all statistical evaluations.

## Results

Between February and May 2013, 134 out of 233 (57.5%; SE = 3.2%) invited referents of Italian medical oncology units agreed to participate to the e-mail survey. Among the initial 134 responses, 125 (93.3%) surveys from oncology departments widely distributed in the Italian territory, were completed. Ninety-seven (77.6%: SE = 3.73) referents state that modalities of breast cancer follow-up are planned according to the risk of disease progression at diagnosis, while 25 (20.0%: SE = 3.58) perform follow-up work similarly for all women. Overall, only 12 (9.6%: SE = 2.63) oncology units apply the minimal follow-up procedures according to international guidelines, 5 units only for patients in the low risk group and 7 units for all patients, not taking into account the risk category. Minimal follow-up is never applied in high risk asymptomatic women. Twenty-three (18.4%: SE = 3.46) oncology units stop surveillance at 5 years of follow-up, 98 (78.4%: SE = 3.78) continue follow-up in all patients beyond 5 years, and 4 (3.2%) continue beyond 5 years only in hormone receptor positive tumors.

### Follow-up modalities according to the risk of disease relapse

**Figure 1:**
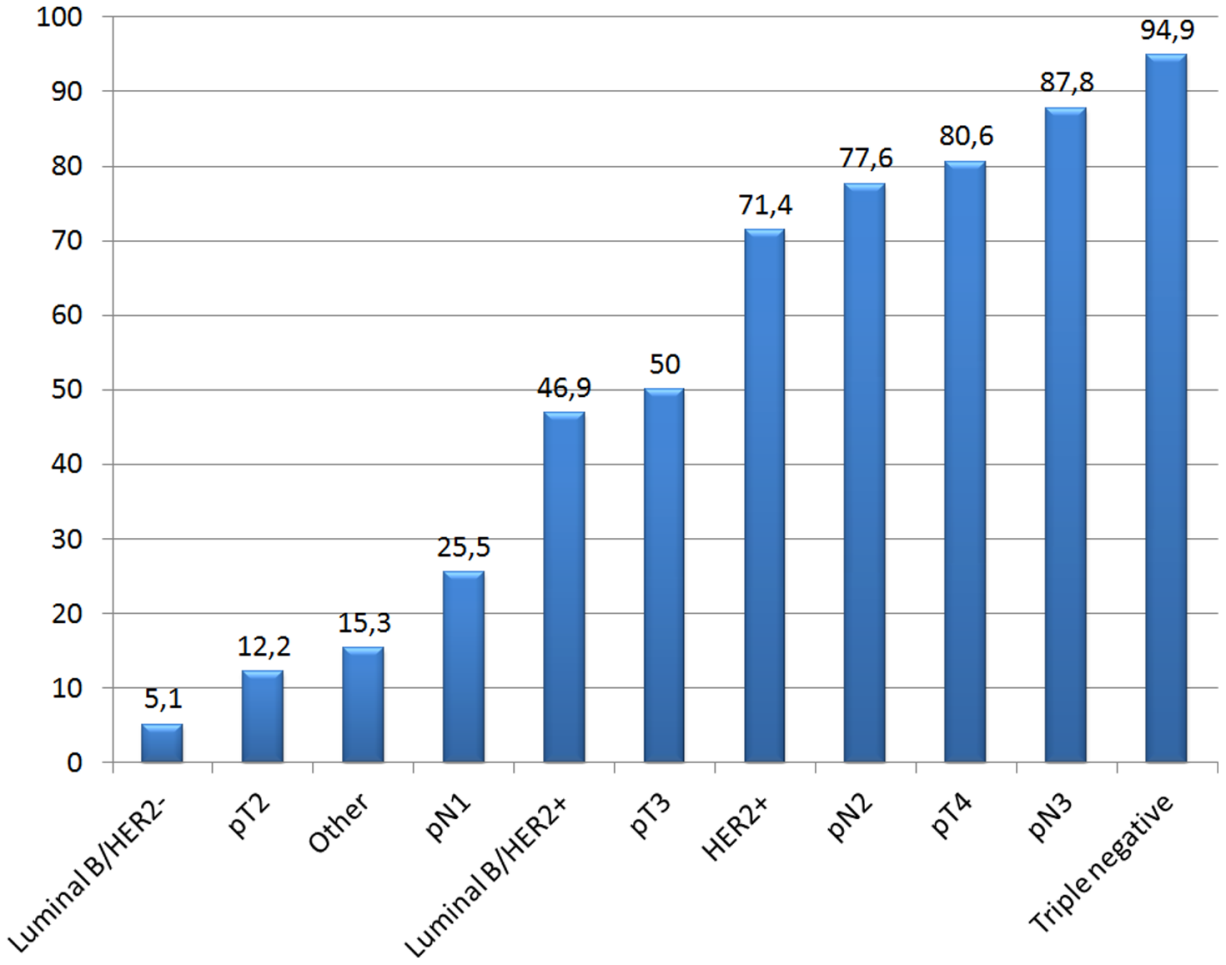
Tumor characteristics considered relevant to classify patients at high risk of disease progression.

Oncology units performing follow-up according to the risk of disease progression were asked to indicate which prognostic factors they deem more relevant to stratify patients in the low and high risk categories. As shown in Fig. 1, more than 70.0% of respondents indicate tumor stage pT4, nodal positivity pN2-pN3, HER2 positivity and triple negativity (i.e. the absence of estrogen, progesteron receptor and HER2 amplification) as the most important factors to classify patients at high risk of disease progression. Other options, not shown, include young age, pre-menopausal status, vascular invasion, high Ki-67 proliferation index, BRCA positivity and familiarity.

Table 1 shows the selection of exams selected at diagnosis according to the risk of disease progression in asymptomatic women. Blood chemistry tests (p = 0.25), chest radiographs (p = 0.23) and liver ultrasounds (p = 0.99) are equally selected for both the low and the high risk groups. Tumor markers are more frequently checked in the high risk group (p = 0.002) as well as bone scans (p = <0.0001). Whole-body CT scan is prescribed by 33.3% of oncologists in the high risk group versus 8.3% in the low risk group (p = <0.0001); similarly, whole-body PET/CT is deemed relevant at diagnosis by 10.7% of oncologists for patient in the high risk group versus 1.1% for the low risk group (p = 0.007).

**Table 1:** Selection of exams at diagnosis and during follow-up according to the risk of disease progression.

	*At diagnosisAt*			*At follow-up*		
	*At diagnosis*					
	*NO*	*YES*	*p value*	*NO*	*YES*	*p value*
	No. (%)	No. (%)		No. (%)	No. (%)	
*Blood chemistry tests*						
Low risk	9 (9.4)	87 (90.6)	0.25	14 (14.6)	82 (85.4)	0.16
High risk	4 (4.2)	92 (95.8)		7 (7.3)	89 (92.7)	
*Tumor markers*						
Low risk	21 (21.7)	76 (78.3)	0.002	15 (15.5)	82 (84.5)	0.03
High risk	6 (6.2)	91 (93.8)		5 (5.5)	92 (94.5)	
*Chest radiograph*						
Low risk	7 (7.3)	89 (92.7)	0.23	36 (37.5)	60 (62.5)	0.005
High risk	12 (13.0)	81 (87.0)		17 (18.3)	76 (81.7)	
*Liver ultrasound*						
Low risk	9 (9.4)	87 (90.6)	0.99	29 (30.2)	67 (69.8)	<0.0001
High risk	8 (8.6)	85 (91.4)		8 (8.6)	85 (91.4)	
*Bone scan*						
Low risk	40 (41.3)	57 (58.7)	<0.0001	69 (73.4)	25 (26.6)	0.006
High risk	3 (3.1)	94 (96.9)		50 (53.2)	44 (46.8)	
*Whole-body CT scan*						
Low risk	88 (91.7)	8 (8.3)	<0.0001	87 (93.5)	6 (6.5)	<0.0001
High risk	64 (66.7)	32 (33.3)		61 (63.6)	32 (34.4)	
*Whole-body PET/CT scan*						
Low risk	94 (98.9)	1 (1.1)	0.007	81 (96.4)	3 (3.6)	0.08
High risk	75 (89.3)	9 (10.7)		75 (89.3)	9 (10.7)	

Selection of exams during follow-up according to the risk of disease progression are also shown in Table 1. Blood chemistry tests are chosen by more than 85% of oncologists (p = 0.16) for both groups of patients, while whole-body PET/CT scanning is not prescribed by most for both categories (p = 0.08). On the other hand, the other exams taken into account are significantly more often selected for patients in the high risk group. However, tumor markers, chest radiograph and liver ultrasound are selected by more than 60% of oncologists also for the low risk group.

### Follow-up modalities independently from the risk of disease relapse

Exams at diagnosis and at follow-up selected independently from the risk of disease relapse are shown in Table 2. Even if numbers are small, more than 87.0% of oncologists prescribe complete staging at diagnosis, with the exception of whole-body CT scan and whole-body PET/CT scan. On the other hand, only blood chemistry tests and tumor markers are selected by 60.0% and 80.0% of oncologists during follow-up, respectively.

**Table 2:** Selection of exams independently of the risk of disease relapse.

	*At diagnosis*		*At follow-up*		
	*NO*	*YES*	*NO*	*YES*	*P value*
	No. (%)	No. (%)	No. (%)	No. (%)	
*Blood chemistry tests*	0	25 (100.0)	10 (40.0)	15 (60.0)	<0.0001
*Tumor markers*	3 (12.0)	22 (88.0)	5 (20.0)	20 (80.0)	0.47
*Chest radiograph*	3 (12.0)	22 (88.0)	18 (72.0)	7 (28.0)	<0.0001
*Liver ultrasound*	0	25 (100.0)	14 (56.0)	11 (44.0)	<0.001
*Bone scan*	1 (4.2)	24 (96.0)	20 (80.0)	5 (20.0)	<0.0001
*Whole-body CT scan*	19 (76.0)	6 (24.0)	25 (100.0)	-	0.01
*Whole-body PET/CT scan*	25 (100.0)	0	25 (100.0)	-	-

### Frequency of exams according to the risk of disease relapse

In the low risk group, as shown in Table 3, blood chemistry tests and tumor markers are prescribed a median of 2 times/year in the first 3 years of follow-up, chest radiographs and liver ultrasound 1 time/year, while bone scan, whole-body CT scan and whole-body PET/CT scan are not taken into account. In the high risk group (Table 3), blood chemistry tests and tumor markers are prescribed a median of 3 times/year in the first 2 years of follow-up, and then 2 times/year. Chest radiograph is prescribed annually for five years, liver ultrasound every six months for the first 2 years and then annually, bone scan annually only for the first 3 years. Whole-body CT scan and whole-body PET/CT scan are not usually prescribed.

**Table 3:** Frequency of exams during follow-up.

	1° year	2° year	3° year	4° year	5° year	P value
	median times/year (range)					
**Low Risk Group**						
*Blood chemistry test*, No. 92	2 (0–4)	2 (0–4)	2 (0–3)	1 (0–2)	1 (0–2)	<0.0001
*Tumor marker*, No. 94	2 (0–4)	2 (0–4)	2 (0–3)	2 (0–3)	1 (0–2)	<0.0001
*Chest radiograph*, No. 80	1 (0–2)	1 (0–2)	1 (0–2)	0 (0–2)	0 (0–2)	<0.0001
*Liver ultrasound*, No. 87	1 (0–2)	1 (0–2)	1 (0–2)	1 (0–2)	1 (0–2)	0.45
*Bone scan*, No. 65	0 (0–1)	0 (0–1)	0 (0–1)	0 (0–1)	0 (0–1)	0.48
*Whole-body CT sca*, No. 46	0 (0–1)	0 (0–1)	0 (0–1)	0 (0–1)	0 (0–1)	0.32
*Whole-body PET/CT scan*, No. 46	0	0 (0–1)	0	0 (0–1)	0	0.41
**High Risk Group**						
*Blood chemistry tests*, No. 92	3 (0–4)	3 (0–4)	2 (0–4)	2 (0–4)	2 (0–4)	<0.0001
*Tumor markers*, No. 94	3 (1–4)	3 (0–4)	2 (0–4)	2 (0–4)	2 (0–4)	<0.0001
*Chest radiograph*, No. 80	1 (0–2)	1 (0–2)	1 (0–2)	1 (0–2)	1 (0–2)	0.45
*Liver ultrasound*, No. 87	2 (0–2)	2 (0–2)	1 (0–2)	1 (0–2)	1 (0–2)	<0.0001
*Bone scan*, No. 65	1 (0–2)	1 (0–2)	1 (0–2)	0 (0–2)	0 (0–2)	<0.0001
*Whole-body CT scan*, No. 46	0.5 (0–2)	0 (0–2)	0 (0–1)	0 (0–1)	0 (0–1)	<0.0001
*Whole-body PET/CT scan, No. 46*	0 (0–1)	0 (0–1)	0 (0–1)	0 (0–1)	0 (0–1)	0.57
***Independently from Risk of Disease Progression***						
*Blood chemistry tests,* No. 19	2 (0–4)	2 (0–3)	2 (0–3)	1 (0–2)	1 (0–2)	<0.0001
*Tumor markers*, No. 20	3 (1–4)	3 (1–4)	2 (1–4)	2 (1–3)	1.5 (1–3)	<0.0001
*Chest radiograph*, No. 14	0.5 (0–2)	0 (0–2)	0 (0–1)	0 (0–1)	0 (0–1)	0.003
*Liver ultrasound*, No. 16	1 (0–2)	1 (0–2)	1 (0–2)	1 (0–2)	1 (0–2)	0.45
*Bone scans*, No. 12	0 (0–1)	0 (0–1)	0 (0–1)	0 (0–1)	0 (0–1)	0.48
*Whole-body CT scan*, No. 10	0	0	0	0	0	-
*Whole-body PET/CT scan*, No. 10	0	0	0	0	0	-

No.: number of responses.

### Frequency of exams independently of the risk of disease relapse

Table 3 shows also that, independently on the risk of progression, blood chemistry tests and tumor markers are frequently prescribed 2 times/year in all patients, liver ultrasound annually while the others are not usually prescribed.

## Discussion

Our survey shows that 90.4% of Italian oncology units who participated in the web-based questionnaire declare they do not apply the minimal breast cancer follow-up procedures after primary treatment in asymptomatic women, as suggested by National and International Oncology Societies [11], [14], [15], [18]. Although participants were almost half of the medical oncology units present in Italy, they were uniformly distributed in the Italian territory (as listed below) and, therefore, the survey may be considered representative of the follow-up preferences of the Italian oncologist. The data confirm a recent retrospective analysis of follow-up care of breast cancer patients by Leoni et al showing that intensive follow-up testing is a quite common clinical practice in the Italian region Emilia-Romagna [24]. These results reflect the never ending, 80 s dating debate on minimal versus intensive follow-up procedures after breast cancer surgery [2], [8], [9], [20], [21], [22], [23], [25], [26] and show that, at least in Italy, minimal follow up procedures are prescribed by a minority of medical oncology units [27]. Similarly, it has been recently reported the use of non-recommended surveillance procedures for early breast cancer patients in a Californian academic medical center [28]. On the contrary, a higher adherence to current guidelines has been reported for most oncologists from other countries, such as USA [20] and Australia [29], [30].

On the other hand, it is the first time, **to** our knowledge, that a high percentage of interviewed referents (about 80.0%), declares to perform exams at diagnosis and follow-up according to the risk of disease progression, high intensity for high risk, low intensity for low risk patients, the so called “tailored follow-up”. Tumor stage pT4, pN2-pN3 and biological factors such as HER2 positivity and triple negativity are indicated as the most relevant prognostic factors to classify patients at high risk of disease progression. These choices are in agreement with literature data showing that pathological stage and intrinsic breast cancer subtypes are the most relevant prognostic factors able to influence clinical outcome [31], [32], [33], [34]. Interestingly, van Hezewijk et al [8], using a web-based 29-item questionnaire, reported that 130 respondents of different disciplines (surgeons, medical oncologists, radiation oncologists and nurse practitioners) identified as patients at high risk to follow-up with a higher frequency of visits those of younger age and with pT3-4/pN2-3 tumor, not taking into account tumor biology, as medical oncologists did in the present study. Other studies, tailoring follow-up according to the risk of disease progression, modulate frequency of visits and overall duration of surveillance, instead of follow-up procedures as Italian oncologists prefer [8], [10], [35], [36]. In the present report tumor markers, bone scan and whole-body CT scan are more frequently prescribed at diagnosis and thereafter in the high risk group, while whole body PET/CT scan is recommended only to stage disease at diagnosis. However, tumor markers, chest radiograph and liver ultrasound are selected by more than half of participants also for the low risk group, and all these modes of surveillance are not recommended by current guidelines [11], [15], [16], [18]. The frequency of follow-up exams is reported to be higher in the first 2–3 years in all groups taken into account, and this is in agreement with data showing a peak of recurrences during the first 2–3 years with a decreasing hazard of disease progression beyond 5 years, even if estrogen dependent tumors may recur many years after initial treatment [37], [38]. Most of participants declare to continue follow-up in all patients, independently from risk category, beyond 5 years, in agreement with international guidelines [9], [15], [16], [18], [26], while very few oncology units continue surveillance beyond 5 years only in hormone receptor positive tumors. Both ASCO and NICE guidelines suggest frequent clinical examination in the first 3–5 years after diagnosis, but after 3 years NICE suggests to discharge patients to general practice while ASCO suggests long-term follow-up [11], [39]. Even if there is certainly an increase in the hazard rate of disease progression in the first 3 years after diagnosis, this peak does not include most of new contralateral cancers. The hazard curves for breast cancer mortality shows an initial increase of ∼3%/year in the rate of distant relapses between the 2^nd^ and the 3^rd^ year of surveillance, with a subsequent fall to ∼2%/year which remains constant for almost 10 years [35], [38]. In contrast, potentially treatable local relapse occurs at a constant rate of 1–1.5% per year for at least 10 years [35], thus hardly justifying discharge at 3 years of follow-up [40], [41]. Moreover, prolonged follow-up care could offer some advantages, i.e. an increased adherence to adjuvant endocrine therapy, known to be higher with long-term follow-up [7], [8], as well as diagnosis and management of long-term toxicities. Late toxic effects of adjuvant treatments may continue for many years, with some patients at increased risk of life-threatening toxicities such as thromboembolic disease, uterine cancer, cerebrovascular or cardiovascular events, second malignancies and more [42], [43].

Finally, we ignore which is the optimal follow-up for extended adjuvant endocrine therapies [44], [45], [46], after treatment with new biological agents [47], as well as the value of a follow-up tailored on distinct patterns of metastatic spread depending on breast cancer subtypes [48]. Moreover, the impact on survival of detecting an oligometastatic disease is still unknown [49], [50] and, hopefully, the utility of an early detection of metastatic disease suitable of cure with the ongoing molecular targeted agents or novel therapeutics drugs [51].

Further research is needed even if ongoing guidelines advise against routine search for distant metastases, since no advantage exists in early diagnosis and treatment [3], [4], [5], [52]. However, considering the plethora of novel active agents that have entered clinical practice for metastatic breast cancer in the last years, randomized clinical trials should be performed to determine the comparative effectiveness of different follow-up modalities, their ideal frequency and duration, and the development of risk-based guidelines [16], [36].

## Supporting Information

Appendix S1 **Members of the “FOLLOW-UP” Study Group.**
(DOCX)
