# Supplementary material for: Breast Cancer “Tailored Follow-up” in Italian Oncology Units: A Web-Based Survey
Source: PLoS One. 2014 Apr 8;9(4):e94063. doi: 10.1371/journal.pone.0094063 (PMC3979748; doi:10.1371/journal.pone.0094063)
Supplement: Appendix S1 — Members of the “FOLLOW-UP” Study Group. (DOCX) [file pone.0094063.s001.docx]

**Appendix I.** Members of the “FOLLOW-UP” Study Group

| Surname and Name | Institution | City | Region |
| --- | --- | --- | --- |
| Natoli Clara  Lead Author  natoli@unich.it | Dipartimento di Scienze Sperimentali e Cliniche, Università "G. D'Annunzio" | Chieti | Abruzzo |
| Adamo Vincenzo | Azienda Ospedaliera Universitaria Policlinico "Gaetano Martino" | Messina | Sicilia |
| Airoldi Mario | Azienda Ospedaliero - Universitaria "San Giovanni Battista" | Torino | Piemonte |
| Amoroso Domenico | Ospedale "Versilia" | Lido di Camaiore | Toscana |
| Angelini Francesco | Ospedale "Regina Apostolorum" | Albano Laziale | Lazio |
| Angiolini Catia | Ospedale "Santa Maria Annunziata" | Firenze | Toscana |
| Angiolucci Giovanni | Ospedale "San Donato" | Arezzo | Toscana |
| Ardizzoia Antonio | Ospedale "Alessandro Manzoni" | Lecco | Lombardia |
| Baldini Editta | Ospedale "Campo di Marte" | Lucca | Toscana |
| Ballardini Pierluigi | Ospedale del Delta | Lagosanto | Emilia Romagna |
| Barni Sandro | Ospedale "Treviglio-Caravaggio" | Treviglio | Lombardia |
| Barone Carlo | Policlinico Universitario "A. Gemelli" | Roma | Lazio |
| Battelli Nicola | Azienda Ospedaliero - Universitaria "Umberto I" | Ancona | Marche |
| Bernardi Daniele | Ospedale Civile | San Donà di Piave | Veneto |
| Bianchetti Sara | Ospedale "Regina Apostolorum" | Albano Laziale | Lazio |
| Bianco Nadia | Policlinico | Monza | Lombardia |
| Biglia Nicoletta | Ospedale Mauriziano "Umberto I" | Torino | Piemonte |
| Bilancia Domenico | Ospedale "San Carlo" | Potenza | Basilicata |
| Biti Gianpaolo | Azienda Ospedaliera Universitaria Careggi | Firenze | Toscana |
| Boni Corrado | Arcispedale "Santa Maria Nuova" | Reggio Emilia | Emilia Romagna |
| Bordonaro Roberto | A.r.n.a.s. Garibaldi - Presidio Ospedaliero Nesima | Catania | Sicilia |
| Botta Mario | Ospedale "Santo Spirito" | Casale Monferrato | Piemonte |
| Bretti Sergio | Presidio Riunito di Ivrea - Cuorgnè - Castellamonte | Ivrea | Piemonte |
| Brunello Antonella | Istituto Oncologico Veneto | Padova | Veneto |
| Brunetti Cosimo | Ospedale "Marianna Giannuzzi" | Manduria | Puglia |
| Bruno Daniele | Azienda Ospedaliera "Gaetano Rummo" | Benevento | Campania |
| Bucci Eraldo | Istituto Ospedaliero Multimedica | Castellanza | Lombardia |
| Buzzoni Roberto | Fondazione I.R.C.C.S. Istituto Nazionale dei Tumori | Milano | Lombardia |
| Cagossi Katia | Ospedale "Ramazzini" | Carpi | Emilia Romagna |
| Cappelletti Claudia | Ospedale "S. Croce" | Fano | Marche |
| Cappuzzo Federico | Ospedale Civile | Livorno | Toscana |
| Cardillo Franca | Presidio Ospedaliero Sud | Gaeta | Lazio |
| Carroccio Rosalia | Presidio Ospedaliero "Umberto I" | Enna | Sicilia |
| Cascinu Stefano | Azienda Ospedaliero-Universitaria "Umberto I" | Ancona | Marche |
| Cavanna Luigi | Ospedale "Guglielmo Da Saliceto" | Piacenza | Emilia Romagna |
| Cianchetti Ettore | Ospedale "G. Bernabeo" | Ortona | Abruzzo |
| Clerico Mario | Ospedale degli Infermi | Biella | Piemonte |
| Contu Antonio | Ospedale Civile "SS. Annunziata" | Sassari | Sardegna |
| Corsi Domenico | Ospedale "San Giovanni Calibita - Fatebenefratelli" | Roma | Lazio |
| Cortesi Laura | Azienda Ospedaliera Policlinico | Modena | Emilia Romagna |
| Cretella Elisabetta | Ospedale centrale | Bolzano | Trentino -Alto Adige |
| Crispino Sergio | Ospedali Riuniti "Valdichiana Senese" | Montepulciano | Toscana |
| Di Lieto Marco | Ospedale "Il Ceppo" | Pistoia | Toscana |
| Di Lullo Liberato | Ospedale "F. Veneziale" | Isernia | Molise |
| Durini Ernesto | Ospedale "Cardinale G. Panico" | Tricase | Puglia |
| Fabi Alessandra | Istituto Nazionale Tumori Regina Elena I.R.C.C.S. - I.F.O. | Roma | Lazio |
| Failla Giuseppe | Centro Clinico e Diagnostico G.B. Morgagni | Catania | Sicilia |
| Fattorusso Silvia | Presidio Ospedaliero Centro | Terracina | Lazio |
| Ferraù Francesco | Ospedale "San Vincenzo" | Taormina | Sicilia |
| Ferro Antonella | Ospedale "Santa Chiara" | Trento | Trentino Alto Adige |
| Ficorella Corrado | Ospedale "San Salvatore" | L'Aquila | Abruzzo |
| Fogazzi Gianluca | Istituto Clinico "S. Anna" | Brescia | Lombardia |
| Foglietta Jennifer | Ospedale "S. Maria della Misericordia" | Perugia | Umbria |
| Francini Guido | Azienda Ospedaliera Universitaria Senese | Siena | Toscana |
| Fusco Ornella | Ospedale civile | Sondrio | Lombardia |
| Gennari Alessandra | E. O. Ospedali "Galliera" | Genova | Liguria |
| Ghiani Massimo | Ospedale Oncologico "Armando Businco" | Cagliari | Sardegna |
| Gianni Lorenzo | Ospedale "Infermi" | Rimini | Emilia Romagna |
| Giordano Monica | Ospedale "Sant'Anna" | Como | Lombardia |
| Giotta Francesco | IRCCS "Giovanni Paolo II" | Bari | Puglia |
| Giuliani Rosa | Ospedale "San Camillo-Forlanini" | Roma | Lazio |
| Gori Stefania | Ospedale "Sacro Cuore - Don Calabria" | Negrar | Veneto |
| Graiff Claudio | Ospedale Centrale | Bolzano | Trentino -Alto Adige |
| Guarneri Valentina | Azienda Ospedaliera Policlinico | Modena | Emilia Romagna |
| Guarneri Domenico | Ospedale Civile "G. Borea" | Sanremo | Liguria |
| Guglielmi Flavio | Ospedale "SS: Annunziata" | Sulmona | Abruzzo |
| Landriscina Matteo | Università degli Studi di Foggia, Dipartimento Scienze Mediche e Chirurgiche | Foggia | Puglia |
| Laudadio Lucio | Ospedale "F. Renzetti" | Lanciano | Abruzzo |
| Lombardo Marco | Ospedale "Spirito Santo" | Pescara | Abruzzo |
| Longo Flavia | Azienda Policlinico Umberto I | Roma | Lazio |
| Macellari Giorgio | Ospedale "Guglielmo Da Saliceto" | Piacenza | Emilia Romagna |
| Madeddu Clelia | Azienda Ospedaliero Universitaria | Cagliari | Sardegna |
| Magnanini Simonetta | Ospedale "San Donato" | Arezzo | Toscana |
| Maiorino Luigi | Ospedale "San Gennaro" | Napoli | Campania |
| Mangiameli Alessandra | Casa di Cura Musumeci | Gravina di Catania | Sicilia |
| Marini Giovanni | Istituto Clinico Sant'Anna | Brescia | Lombardia |
| Massidda Bruno | Azienda Ospedaliero Universitaria | Cagliari | Sardegna |
| Mattioli Rodolfo | Ospedale "S. Croce2 | Fano | Marche |
| Michelotti Andrea | Azienda Ospedaliero-Universitaria Pisana | Pisa | Toscana |
| Molino Annamaria | Azienda Ospedaliera Universitaria Integrata | Verona | Veneto |
| Montesarchio Vincenzo | Azienda Ospedaliera "Domenico Cotugno" | Napoli | Campania |
| Morale Antonella | Ospedale "Mazzoni" | Ascoli Piceno | Marche |
| Murgo Roberto | IRCCS Ospedale "Casa Sollievo della Sofferenza" | San Giovanni Rotondo | Puglia |
| Naso Giuseppe | Azienda Policlinico Umberto I | Roma | Lazio |
| Natale Donato | Ospedale "S. Massimo" | Penne | Abruzzo |
| Orditura Michele | Azienda Ospedaliera Seconda Università Degli Studi | Napoli | Campania |
| Orrù Sandra | Ospedale Oncologico "A. Businco" | Cagliari | Sardegna |
| Pace Roberta | Ospedale "S. Camillo de' Lellis" | Rieti | Lazio |
| Palazzo Antonella | Azienda Policlinico Umberto I | Roma | Lazio |
| Palma Fedele | Ospedale "Antonio Perrino" | Brindisi | Puglia |
| Pancotti Amedeo | Ospedale Civile "Giuseppe Mazzini" | Teramo | Abruzzo |
| Pandoli Giuliano | Ospedale "Spirito Santo" | Pescara | Abruzzo |
| Papaldo Paola | Istituto Nazionale Tumori Regina Elena I.R.C.C.S. - I.F.O. | Roma | Lazio |
| Parisi Anna Maria | Ospedale "S.Camillo-Forlanini" | Roma | Lazio |
| Passalacqua Rodolfo | Istituti Ospitalieri | Cremona | Lombardia |
| Pellegrino Arianna | Ospedale "San Pietro" | Roma | Lazio |
| Perrucci Bruno | Istituti Ospitalieri | Cremona | Lombardia |
| Proietti Emanuela | Ospedale "San Giovanni Calibita - Fatebenefratelli" | Roma | Lazio |
| Recchia Francesco | Ospedale "SS. Nicola e Filippo" | Avezzano | Abruzzo |
| Riccardi Ferdinando | AORN "Cardarelli" | Napoli | Campania |
| Rispoli Anna Iolanda | Azienda Ospedaliera Universitaria Careggi | Firenze | Toscana |
| Rocca Andrea | IRCCS-IRST | Meldola | Emilia Romagna |
| Romaniello Incoronata | Presidi Ospedalieri Riuniti | Borgomanero | Piemonte |
| Rossetti Riccardo | Presidio Unificato AUSL 2 | Perugia | Umbria |
| Rossi David | Ospedale " S. Salvatore" | Pesaro | Marche |
| Rosti Giovanni | Ospedale "San Maria di Cà Foncello" | Treviso | Veneto |
| Ruggeri Enzo Maria | Complesso Ospedaliero "Belcolle" | Viterbo | Lazio |
| Russo Antonio | Azienda Ospedaliera Universitaria Policlinico "Paolo Giaccone" | Palermo | Sicilia |
| Savarino Antonino | Ospedale "Barone Lombardo" | Canicattì | Sicilia |
| Savastano Clementina | Azienda Ospedaliera Universitaria "San Giovanni di Dio e Ruggi D'Aragona" | Salerno | Campania |
| Scognamiglio Giovanni | Ospedale Valduce | Como | Lombardia |
| Scognamiglio MariaTeresa | Ospedale”G. Barnabeo” | Ortona | Abruzzo |
| Seminara Patrizia | Azienda Policlinico Umberto I | Roma | Lazio |
| Serrachini Silvia | Azienda Ospedaliera "S. Maria degli Angeli" | Pordenone | Friuli Venezia Giulia |
| Sidoti Vincenzo | Ospedale Civile "Edoardo Agnelli" | Pinerolo | Piemonte |
| Silva Rosa Rita | Ospedale "Egles Profili" | Fabriano | Marche |
| Surace Giuseppe | Presidio Ospedaliero di Ostuni - Fasano - Cisternino | Ostuni | Puglia |
| Tomao Silverio | Ospedale "S. Maria Goretti" | Latina | Lazio |
| Tonini Giuseppe | Università Campus Biomedico | Roma | Lazio |
| Trenta Patrizia | Azienda Policlinico Umberto I | Roma | Lazio |
| Turazza Monica | Ospedale "Sacro Cuore - Don Calabria" | Negrar | Veneto |
| Valenza Roberto | Azienda Ospedaliera "Vittorio Emanuele" | Gela | Sicilia |
| Veltri Enzo | Presidio Ospedaliero Centro | Latina | Lazio |
| Zampa Germano | Ospedale "Regina Margherita" | Roma | Lazio |
| Zaniboni Alberto | Fondazione Poliambulanza - Istituto Ospedaliero | Brescia | Lombardia |
| Zanirato Sonia | AO Pavia - Ospedale Civile Vigevano | Pavia | Lombardia |
